# Supplementary material for: Cause-effect perception in an object place task
Source: Front Cognit. 2026 Jan 2;4:1565294. doi: 10.3389/fcogn.2025.1565294 (PMC13281106; doi:10.3389/fcogn.2025.1565294)

1 ON THE BAYESIAN CAUSAL INFERENCE MODEL OF HUMAN PERCEPTION

There is a fundamental difference between the structural discovery approach suggested here and Bayesian Causal Inference (Bayesian CI) model of multisensory perception and sensorimotor adaptation (Körding et al., 2007; Shams and Beierholm, 2022). In a strict sense, Bayesian CI is not a causal discovery method. It works on a single trial whereas our approach makes use of the statistics of a set of data (from multiple trials of the experiment). Furthermore, in Bayesian CI there are two prespecified hypotheses for the causal structure which have been *previously* learned and are then used by BCI. Shams and Beierholm (2022) state, it is assumed that "The competing causal structures [...] have been learned by experience or over the course of evolution". Therefore, Bayesian CI describes, how the system makes use of the competing causal structures once they have been established (e.g. by some form of causal structure learning). Whereas in causal discovery there are multiple causal structures (the set of all causal structures that are possible with the given set of variables) and by learning from data it has to be determined which of them is the correct one. In our approach, the underlying causal structure is not assumed to vary from trial to trial but is assumed to be (approximately) fixed throughout a period of the experiment. Its identification requires a sufficiently large set of data and cannot be obtained from the data of a single trial. Our method is a classical causal discovery approach in the sense that we have available a set of covariating values of causal variables (data from the trials of one or more sessions of our experiment) and try to identify the underlying causal structure.

2 EXPERIMENT TRIAL DESIGN

The VR setting is made up of two plates, and indicator bar, and a closed hand holding a glass of wine as depicted in fig. 4b. A trial consists of placing the glass on the right plate, moving it on the left plate and touch the bar at the top at some point in between.

The subject is instructed not to break the glass and to perform the states *Transport Pre* and *Transport Post* (see item 3 and item 4) as fast as possible. The glass will break if the variable *result* in the causal model becomes zero, i.e. the haptic device registers a force above a given threshold (see **Causal relations**).

To guide the subject, the next target to touch (plate/bar) appears blue until touched. Once it has touched by the glass, the start (right) plate will first become yellow then green, to indicate the start of the trial. The end (left) plate will act equivalent before the subject raises the glass to end the trial. The whole trial can be seen as the sequence of the following states:

1. **Inter Trial** The glass is weightless. The subject can move the glass freely. The start plate becomes blue.
2. **Touching Start Plate** The glass is placed on top of the start plate, which in turn first becomes yellow, then green. The bar at the top becomes blue once the glass does not touch the plate anymore.
3. **Transport Pre** The glass has to touch the bar at the top, which turns yellow once touched.
4. **Transport Post** The glass has to touch the end plate.
5. **Touching End Plate** The glass is placed on top of the end plate, which in turn first becomes yellow, then green. Afterwards the subject has to stop touching the plate. The glass breaks if the subject applies too much force during this state.
6. **End** The glass becomes weightless and the subject gets feedback about the used force and whether the trial was successful or not.

3 CAUSAL RELATIONS

The causal relations that govern the relevant features in the simulation are given by eqs. (3.1) to (3.4) (note that the prefix *glass* has been omitted for readability). See **??** to for a better overview of the causal information flow between the variables.

The exogenous features are sampled as

$$\begin{array}{lll} \textit{force} & & \text{rendered force by } \textit{PHANToM} \text{ in Newton} \\ \textit{color} & \sim & \mathcal{U}(\{\text{red, green, blue}\}) \\ \textit{weight} & \sim & \mathcal{U}([0.2616, 1]) \end{array} \tag{3.1}$$

The weight is rendered by the haptic device as a force vector pointing directly downwards and is therefore able to simulate the weight of the object. The actual force acting on the subject in Newton is given by $weight \cdot 3N$. *force_threshold* and subsequently *result* are calculated as

$$f_{\textit{force\_threshold}}(\textit{color}, \textit{weight}) = (2.5 \cdot \textit{weight} + \textit{color\_offset}(\textit{color})) \cdot 0.8241 \tag{3.2}$$

and

$$f_{\textit{result}}(\textit{force\_threshold}, \textit{force}) = \begin{cases} 1, & \text{for } \textit{force} \leq \textit{force\_threshold} \\ 0, & \text{else,} \end{cases} \tag{3.3}$$

with

$$\textit{color\_offset}(\textit{color}) = \begin{cases} -0.254 & \text{for } \textit{color} = \text{red} \\ -0.069 & \text{for } \textit{color} = \text{green} \\ 0.116 & \text{for } \textit{color} = \text{blue} \\ . \end{cases} \tag{3.4}$$

If $f_{\textit{result}}(\textit{force\_threshold}, \textit{force}) = 1$, the glass remains intact. Otherwise, it breaks. One can imagine the relation as

$$\text{Glass breaks} = 1 - f_{\textit{result}}(\textit{force\_threshold}, \textit{force}). \tag{3.5}$$

The color-specific offsets for *force_threshold* and the functional form were empirically determined to produce a noticeable difference in the breakability of the glass for each color without being too obvious. After we ran the study with two test participants, we had to correct the threshold in eq. (3.2) by a factor of 0.8241 because the glass was too sturdy. We verified the design with one last test participant.

4 CAUSAL DISCOVERY RESULTS

Whenever we applied causal discovery to data, we used the FCI, PC and FGES algorithms. All results we report are similar based on the different algorithms using the specified criteria as can be seen in the following figures.

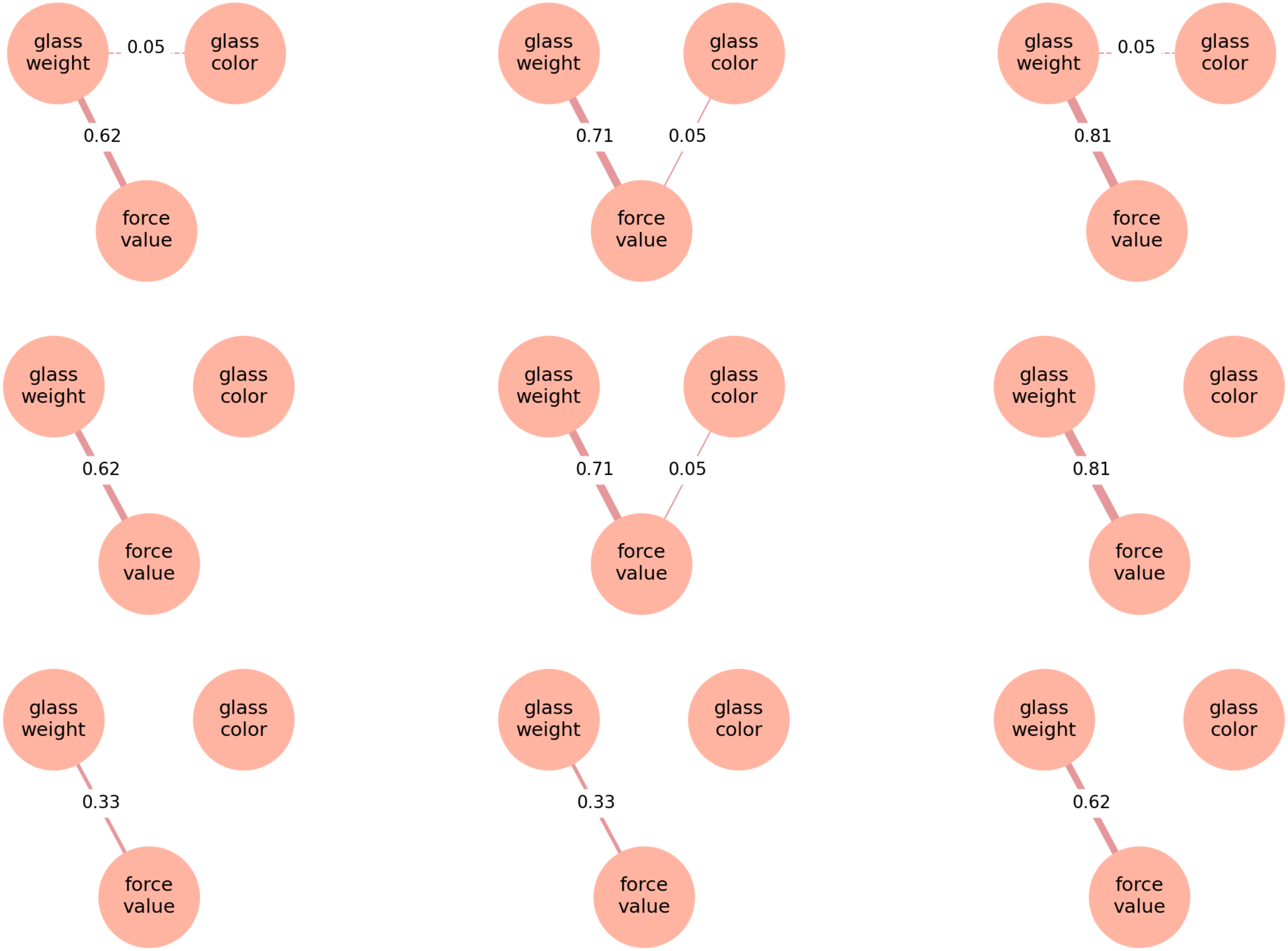

Figure 16. Sensorimotor causal graphs identified by the causal discovery algorithm (sessions from left to right: raw, train, test, algorithms from top to bottom: FCI, PC, FGES)

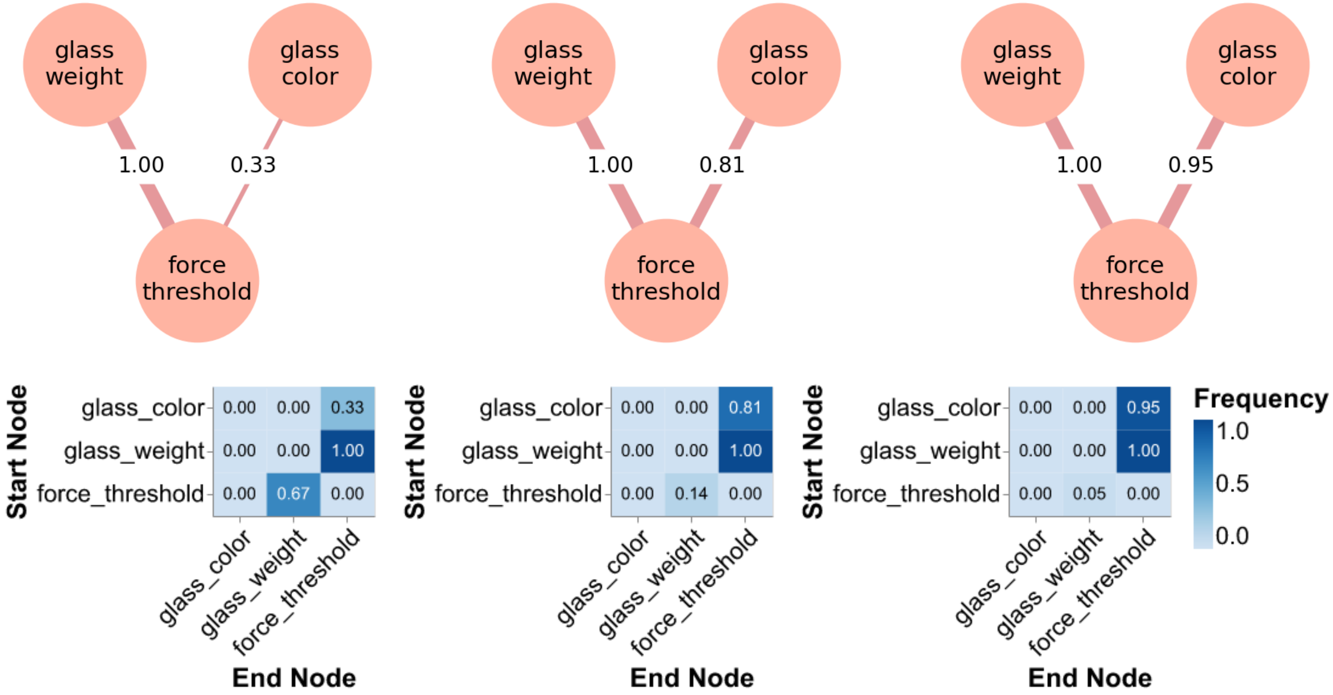

Figure 17. Causal machine learning with discovery algorithms across the three sessions with an idealized variable set for FCI algorithm. Upper part: Causal connectivity. Lower part: Causal directions.

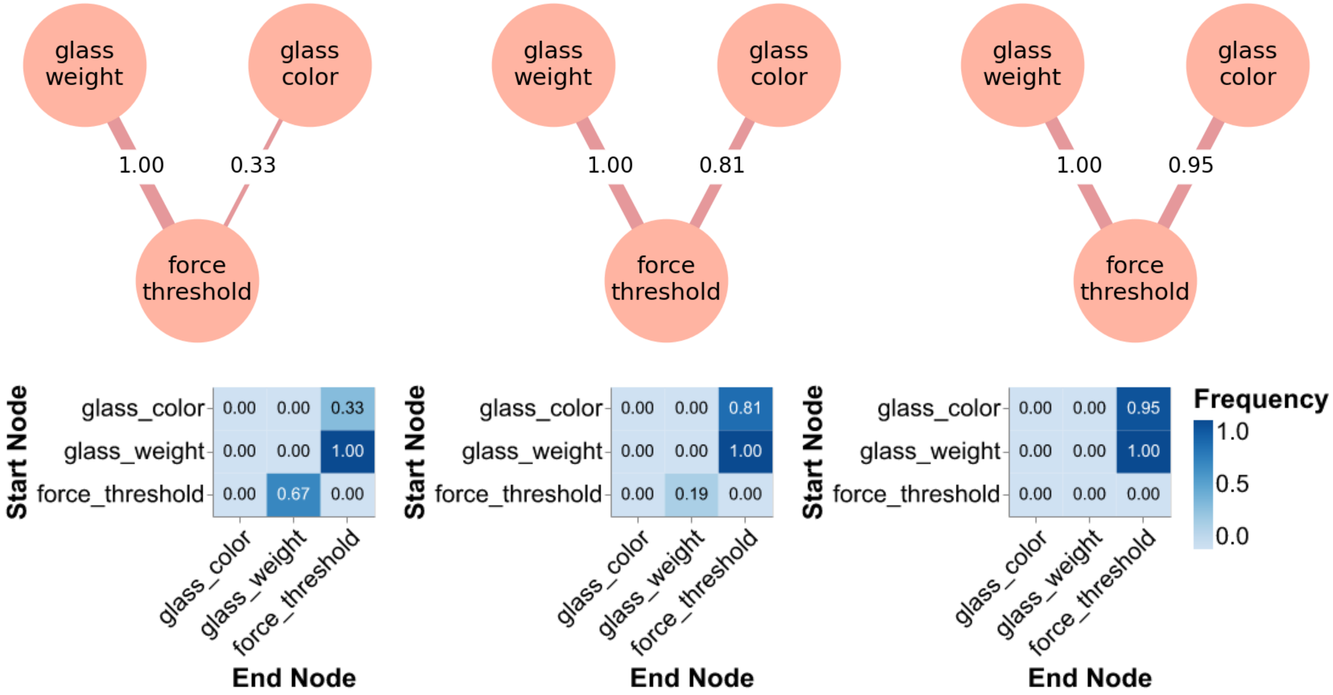

Figure 18. Causal machine learning with discovery algorithms across the three sessions with an idealized variable set for PC algorithm. Upper part: Causal connectivity. Lower part: Causal directions.

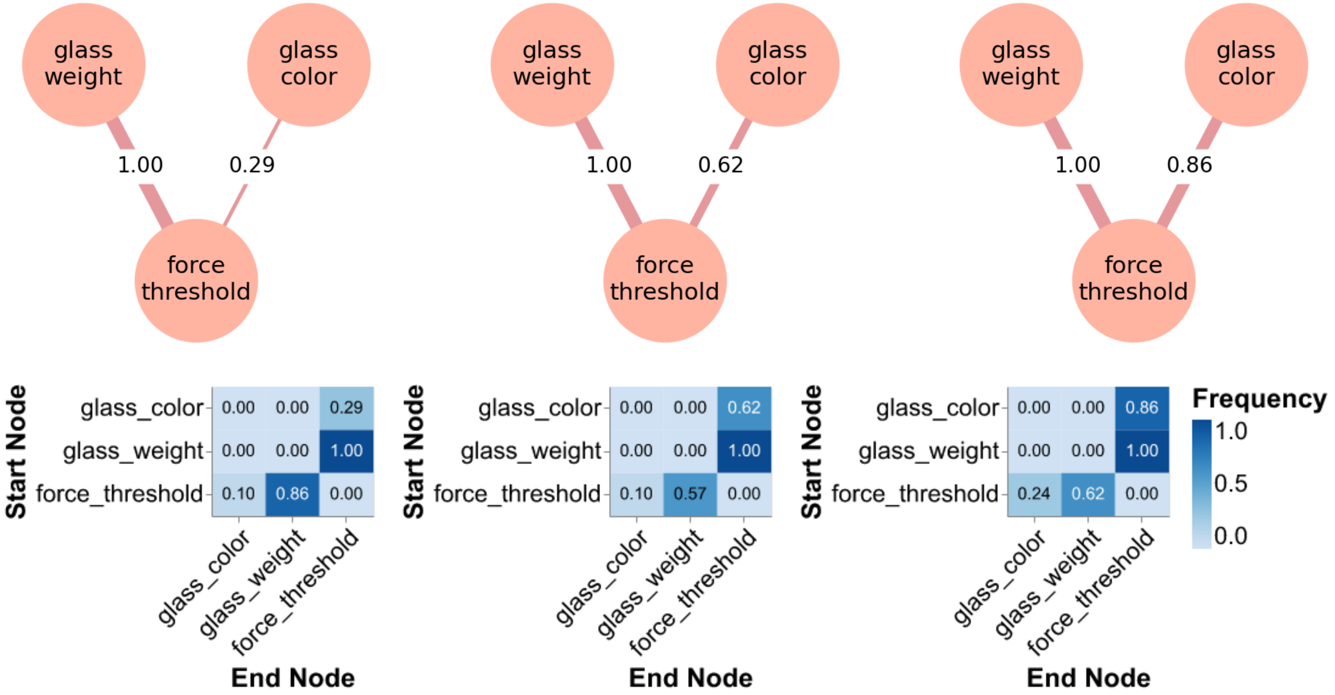

Figure 19. Causal machine learning with discovery algorithms across the three sessions with an idealized variable set for FGES algorithm. Upper part: Causal connectivity. Lower part: Causal directions.

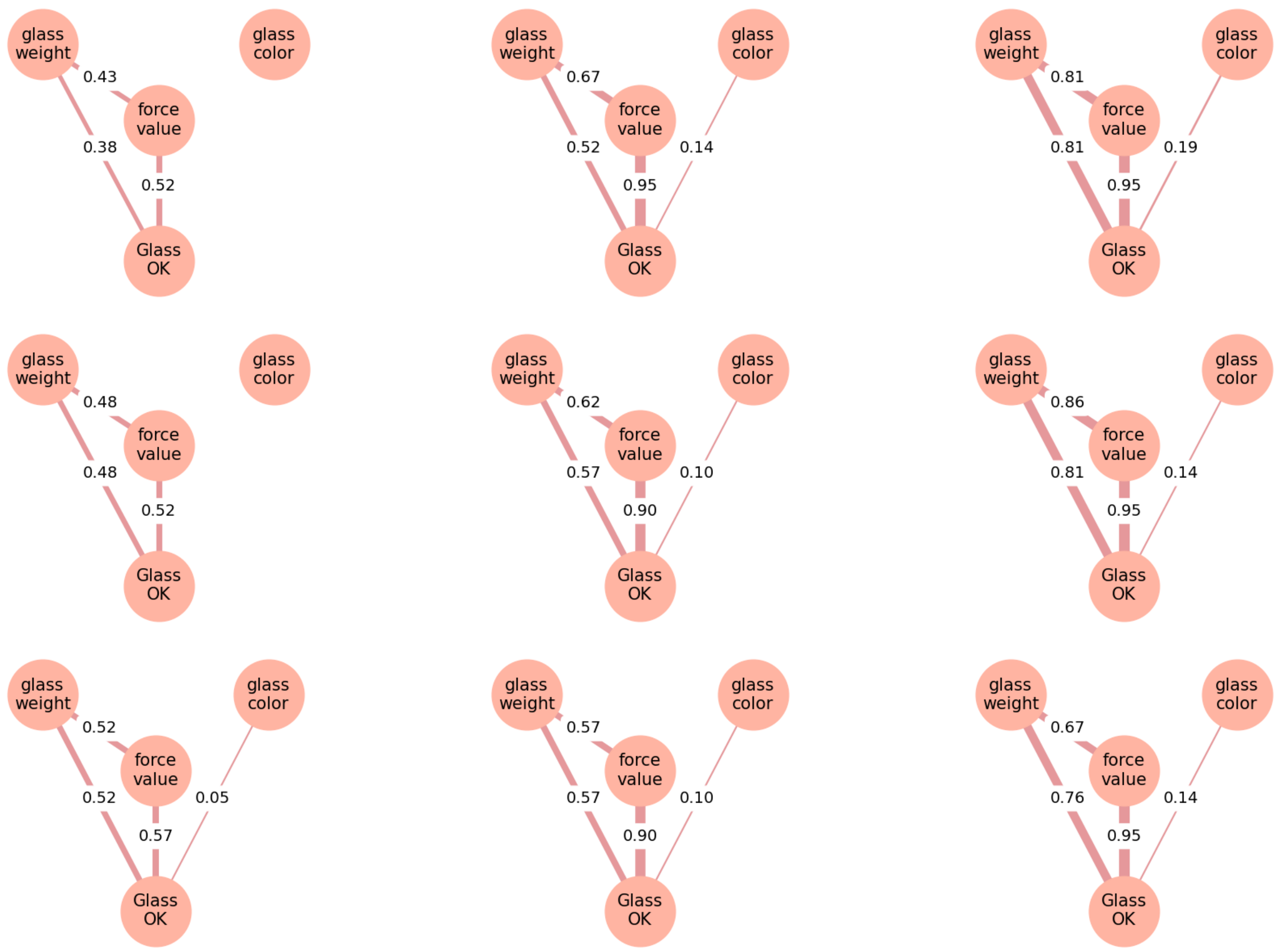

Figure 20. Causal relations discovered by a machine algorithm from the observable data available to humans in the experiment (sessions from left to right: raw, train, test, algorithms from top to bottom: FCI, PC, FGES)

5 PARAMETERS

Parameter	Value
Model type	Phantom Premium 1.5
Device version	3.50.0
Driver version	5.1.7
Firmware version	12
Force ramping rate	0.5 [N/s]
Update rate	1000 [Hz]
Stiffness	1
Static friction	0
Dynamic friction	0

Table 1. Specifications and parameter used for the Phantom haptic device

Algorithm	Hyperparameter	value
	resamplingWithReplacement	yes
	addOriginalDataset	yes
	saveBootstrapGraphs	no
	percentResampleSize	80
	numberResampling	60
	resamplingEnemble	1
	seed	-1
	timeLag	0
	stableFAS	yes
	discretize	yes
	numCategoriesToDiscretize	3
	alpha	0.01
	depth	-1
	maxPathLength	-1
FCI	completeRuleSetUsed	yes
FCI	doDiscriminatingPathRule	yes
FCI	possibleMsepDone	yes
PC	meekPreventCycles	yes
PC	useMaxPHeuristic	no
PC	conflictRule	1

Table 2. Used hyperparameter for the FCI and PC algorithm. The column *Algorithm* denotes a value/hyperparameter, which is only set for specific criteria. If left empty, the setting is used for both algorithms.

**Hyperparameter**	**value**
resamplingWithReplacement	yes
addOriginalDataset	yes
saveBootstrapGraphs	no
percentResampleSize	80
numberResampling	60
resamplingEnsemble	1
seed	-1
timeLag	0
faithfulnessAssumed	no
meekVerbose	no
parallelized	no
precomputeCovariances	yes
symmetricFirstStep	no
structurePrior	1.0
maxDegree	1000
penaltyDiscount	2.0

Table 3. Used hyperparameter for the FGES algorithm

6 QUESTIONNAIRE (GERMAN)

Fragen

- Glas-Farbe beeinflusst

☐ Glas-Gewicht ☐ Glas-Zerbrechlichkeit ☐ Nichts

- Glas-Gewicht beeinflusst

☐ Glas-Farbe ☐ Glas-Zerbrechlichkeit ☐ Nichts

- Glas-Zerbrechlichkeit beeinflusst

☐ Glas-Gewicht ☐ Glas-Farbe ☐ Nichts

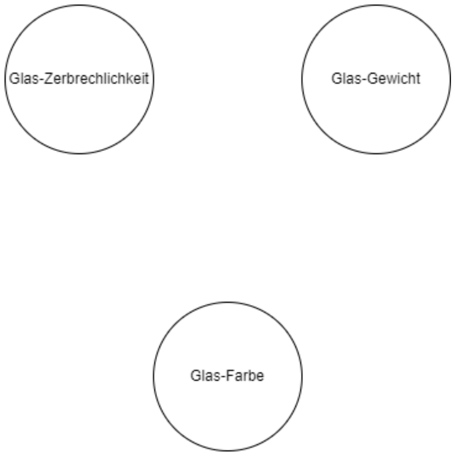

Supplement: Supplementary file 1 [file Data_Sheet_1.pdf]
